# Chorea and Cognitive Impairment in JAK2V617F-Positive Myeloproliferative Disorders: A Case Report and Literature Review

**DOI:** 10.3390/medicina60010018

**Published:** 2023-12-21

**Authors:** Ioana Butnariu, Dana Antonescu-Ghelmez, Adriana Moraru, Daniela Nicoleta Anghel, Florentina Melania Cojocaru, Sorin Tuță, Adela Magdalena Ciobanu, Florian Antonescu

**Affiliations:** 1 Department of Clinical Neurosciences, “Carol Davila” University of Medicine and Pharmacy, 020023 Bucharest, Romania; 2 Neurology Department, National Institute of Neurology and Neurovascular Diseases, 041915 Bucharest, Romania; 3 “Prof. Dr. Alexandru Obregia” Clinical Psychiatry Hospital, 041914 Bucharest, Romania

**Keywords:** chorea, movement disorders, primary myelofibrosis, myeloproliferative disorders, JAK2 V617F

## Abstract

Chorea is a hyperkinetic movement disorder, accompanied by dystonia, myoclonus, tics, stereotypies, and tremors. It is characterized by excessive, purposeless movements that are distressing, irregularly timed, and randomly distributed. Chorea can be present in many diseases, such as hereditary, metabolic disturbance, drug-induced, and functional disorders, and, rarely, genetic, autoimmune, and infectious diseases. Primary myelofibrosis (PMF) is a myeloproliferative neoplasm that leads to ineffective clonal hematopoiesis, fibrous tissue deposits in the bone marrow, extramedullary hematopoiesis, and splenomegaly. In rare cases, following uncertain pathological mechanisms, it can present with chorea, particularly affecting the limbs, head, and orofaciolingual muscles. We present a case of a male patient with evolving PMF over several years who was admitted for progressive cognitive impairment and generalized involuntary movement disorder. We also present a review of all cases of myeloproliferative disorders presenting with chorea published in the last 40 years.

## 1. Introduction

Janus Kinase 2 (JAK2) is a gene that promotes the production of blood cells from hematopoietic stem cells and controls the signal transduction of the erythropoietin, thrombopoietin, and related receptors that regulate erythrocyte and megakaryocyte expansion [1,2]. A gain-of-function mutation in this gene (most commonly JAK2 V617F) can cause at least three myeloproliferative disorders (MPDs) including polycythemia vera, primary myelofibrosis (PMF), and essential thrombocythemia [2,3,4]. PMF is the least frequent of the three, with an estimated annual incidence of 0.5–1.5 per 100,000, affecting mainly the over 50 years age group, with a median age at presentation of 67 years and with an equal distribution of men and women [5,6,7]. PMF is characterized by clonal ineffective hematopoiesis with the dysregulation of the normal production of blood cells and can present with an increased white cell count, thrombocythemia or thrombocytopenia, and anemia. In addition, extramedullary hematopoiesis, overstimulated fibroblasts in abnormal microenvironments, and reactive bone marrow with fibrous tissue and reticulin deposits could be involved. In some cases, PMF can progress to acute leukemia [5,8,9]. In 2008, the World Health Organization (WHO) included the prefibrotic phase of primary myelofibrosis (prePMF) in the classification of myeloid neoplasms, as the prodromal phase of PMF is characterized by hypercellularity, with an increased number of neutrophils but also atypical megakaryocytes and thrombocytosis, so that only a bone marrow biopsy could distinguish between prePMF and ET [10].

Chorea is a hyperkinetic movement disorder, described as involuntary, repetitive, and irregularly timed movements with a generalized or focal distribution [11]. Seldomly, it can occur as a complication of some myeloproliferative neoplasms. In our review of the literature, there are some cases of polycythemia vera, a few essential cases of thrombocythemia, and only one case of secondary myelofibrosis with associated chorea [1,2,4,11,12,13,14,15]. We present a rare case of chorea with progressive cognitive impairment in a patient with a history of JAK2V617F prePMF.

## 2. Case Report

We present the case of a 58-year-old male patient with a two-year history of slowly progressive cognitive impairment and choreiform movements affecting predominantly the upper limbs and orofaciolingual muscles. Seventeen years earlier, he had developed severe splenomegaly and was diagnosed with the hypercellular prefibrotic stage of myeloid metaplasia with myelofibrosis and was positive for the JAK2V617F homozygous genotype, confirmed via bone marrow biopsy. The BCR::ABL1 gene expression was negative and he had grade 5 splenomegaly on Hackett’s scale. The patient had received hydroxyurea 500 mg once daily from 2009 to 2015 when he decided to interrupt its administration despite medical recommendations. Even in the absence of treatment, his hematological disorder remained stable.

Neurological examination revealed involuntary choreiform movements mainly affecting the head and the arms with oral and facial dyskinesia. The gait was ataxic. The movements ceased during sleep. Muscle strength, sensation, and deep tendon reflexes were normal. There was no parkinsonism, dystonia, or pyramidal signs. The patient was slightly hypotonic. The general exam only noted splenomegaly.

Alongside the movement disorder, the patient had developed cognitive problems with memory, attention, and language deficits. These had recently become severe and were accompanied by hypersomnia, depressive mood with a tendency for self-isolation, hyperphagia, and refusal of treatment.

Additionally, three months earlier, the patient presented a psychotic episode with a suicidal attempt by defenestration. The patient had previously worked as a tram conductor, but due to cognitive decline, he left his job approximately one year earlier. There was no history of other medical conditions or exposure to antiemetics, antiparkinsonian, antipsychotic, or neuroleptic drugs. His family history was unremarkable.

A Montreal cognitive assessment (MoCA) was performed, scoring 15/30, with impairment in all cognitive domains. The most affected areas were visuo-spatial orientation, abstraction, language, and calculus, although temporo-spatial orientation was not affected. Memory was moderately affected. A detailed psycho-cognitive assessment was conducted, further highlighting concentrative hypoprosexia in fixation, reproduction, and evocation of presented stimuli. Additionally, false evocations, poor lexical and informational volume lacking conceptual coverage, dysarthric-dyslexic-like speech disorders, dyslexia, dysgraphia, dyscalculia, and apraxia were observed.

A routine blood test at admission revealed a slightly elevated INR and prothrombin time (INR 1.61, PT 21.3 s), no changes in red or white cell lines, a normal platelet count, and no changes in hemoglobin or hematocrit (Table 1). Thyroid function, serum ammonia, and inflammatory markers were within normal limits. He tested negative for HIV. During hospitalization, the INR was repeatedly reassessed, with the persistence of slightly elevated values (1.61–2.07), without correction after vitamin K supplementation. We intended to test the CSF for tau and B-amyloid, but the increased INR prevented us from performing a lumbar puncture. Genetic testing for Huntington’s disease was negative. The cerebral magnetic resonance imaging showed moderate fronto-parietal-insular brain atrophy, with relative sparing of the hippocampal region, minimal cerebellar atrophy, and some chronic supratentorial microangiopathic demyelinating lesions. No lesions were observed in the basal ganglia or diencephalic structures. He was referred for hematological reassessment and treatment with hydroxyurea (500 mg once daily), and folic acid was reinstated. The psychiatric evaluation recommended treatment with sertraline and memantine, which were well tolerated. At the 3-month and 10-month follow-ups, the choreiform movements were remitted, although his family members still reported noticing them occasionally, with the persistence of cognitive impairment (MoCA test 16/30 at the 3-month follow-up and, 17/30 at the 6-month follow-up) and coagulation abnormalities (INR 1.9-2.44) without any reports of bleeding.

## 3. Discussion

### 3.1. The Link between MPD and Chorea

The etiology of PMF is still unknown, but it is generally thought to be genetic, caused by a mutation in JAK2 (JAK2V617F), which is present in 60–65% of these patients [5]. JAK2 is a non-receptor tyrosine kinase involved in multiple signaling pathways [16]. One of these is JAK2/STAT3, a universal intracellular signaling pathway with an essential role in cell proliferation, differentiation, and apoptosis, and also in hematopoiesis and the regulation of the immune system [17]. Aberrant activation of this pathway is commonly detected in multiple types of solid and hematological cancers [17]. JAK2 is also expressed in vivo by striatal progenitor cells in the central nervous system [18]. The JAK2V617F mutation disturbs JAK2′s auto-inhibitory activity, leading to increased neuroinflammation, astrogliosis, and the intensification of cell proliferation, differentiation, and migration [13,18,19].

The JAK-STAT pathway also ensures the homeostasis of the vascular endothelium [20]. Starting from this hypothesis, a recent study compared the mutational profiles of hematopoietic progenitor cells and circulating endothelial cells in patients with PMF. It showed that the JAK2 mutation is present in both cell populations, suggesting that endothelial circulating cells could play an important role in the pathogenesis and progression of PMF, as well as the associated vascular complications [21].

The increased blood cell number represents a risk factor for hyperviscosity and thrombosis [18,19]. This prothrombotic state, leading to ischemic lesions in the basal ganglia, can cause chorea, a neurological complication described especially in PV, with a prevalence of 1–2.5% [22]. It can manifest as acute-onset, sometimes reversible hemichorea or progressive generalized chorea, particularly involving the oromandibular muscles [14].

Although this vascular hypothesis is the most accepted, the etiology of chorea in the MPD is far from being elucidated, as not all patients presenting with movement disorders have a high hematocrit or ischemic lesions of the basal ganglia on an MRI. Furthermore, chorea is not frequent in MPD, not even in patients with symptoms of hyperviscosity in other organs [14,18]. Unexpectedly, functional neuroimaging and pathological studies have, until now, failed to find differences in the neostriatal structures of patients presenting with or without chorea in PV [14]. Therefore, with the current level of evidence, hematological abnormalities, hyperviscosity, and cerebral hypoperfusion can explain only partially the presence of chorea [13]. Additionally, hyperviscosity is present in multiple myeloma and Walderstrom’s macroglobulinemia, but, until now, there are no reported cases associating this with chorea [2]. A possible explanation could be that not all cases of hyperviscosity are similar. In paraproteinemias, the hyperviscosity is caused by the content of important proteins in the plasma, while in MPD, it is secondary to the increased cellular fraction [11]. Still, chorea is sometimes responsive to treatment with hydroxyurea and a reduction in cell numbers, as in the case of our patient.

Another proposed mechanism for chorea is a direct effect of the JAK2V617F mutation, postulating a chronic neuroinflammatory effect, with the cytokinic hyper-activation of the neostriatum [14]. Other studies have suggested dopamine circuit dysfunction as a potential mechanism for chorea: either through increased dopamine receptor sensitivity in the context of reduced levels of catecholamines and serotonin, increased dopamine release in the platelets and its accumulation in the basal ganglia, or the altered metabolic turnover of dopamine in the striatum [2,13,14]. The latter mechanism could be sustained by the response to tetrabenazine [13].

### 3.2. Reviewing the Available Literature

In our literature review summarized in Table 2, we included all cases of primary MPD in adults complicated with chorea, excluding those secondary to cardiac or hormonal pathology. Literature was included if published in English and French within the last 40 years.

Altogether, we identified 24 cases, of which 6 were men and 18 were women. Nineteen patients had PV, three had ET, and two cases of post-PV myelofibrosis were included, of which one reverted to PV. As far as we could tell, no case of PMF has been published yet.

Only in a minority of cases did the MPD diagnosis precede the onset of a movement disorder. Two of the patients with PV had their diagnoses already established approximately 10 years earlier. This was also the case in two of the three patients with ET. Notably, one of them developed chorea after self-stopping the cytoreducing treatment [13].

Thirteen patients had generalized chorea, seven had hemichorea, two had chorea only in one limb, two patients had chorea only in the upper limbs, and nineteen had involuntary movements in the face. Dysarthria was also described in one-third of patients.

Organomegaly was expressed according to the information obtained from the clinical or paraclinical data (abdominal computer tomography or ultrasound) as splenomegaly and/or hepatomegaly. In only one case of PV and one case of post-PV MF did the patients have splenomegaly and hepatomegaly, while in one case of PV, splenomegaly was exclusive [15,23,24]. In two cases, there was no information in this direction, and the other nineteen patients had no organomegaly.

Regarding blood viscosity, it was explicitly mentioned as increased in only two articles [15,23].

Only half of the patients had a bone marrow biopsy. Of these, one patient had normal marrow, eleven had hyperplasia, which in ten of the cases, involved all hematogenous lines (some with a predominance of one or two of them), and in the remaining case, hyperplasia affected only the erythrocyte line.

Nine patients were genetically tested for Huntington’s disease, and all of them were negative. Eighteen patients were tested for the JAK2 mutation, and all were positive. Most cases that were not tested were published before 2000 [15,25,26].

All patients had cerebral imaging (seventeen underwent a cerebral MRI, seven underwent a cerebral CT scan, and one underwent SPECT and PET scans). Three patients had chronic lesions at the level of the basal ganglia and eight patients had cerebral small vessels disease (Table 2).

Response to treatment was expressed as improvement/remission/no recurrence of chorea and as improvement/normalization for the hematological tests. Half of the patients received hydroxyurea: five patients exclusively received hydroxyurea, nine patients received hydroxyurea without phlebotomy, three patients received hydroxyurea and phlebotomies, and two patients were also treated with tetrabenazine. Fourteen of the patients were phlebotomized. All the patients improved clinically and hematologically, except for one case, in which only the hematological abnormalities improved, but the chorea persisted, suggesting that the mechanism by which chorea appeared was not exclusively explained by hyperviscosity [1]. This leads to the idea that either hydroxyurea has an intrinsic effect on choreic movements or that there are other metabolic deficits that are not visible on the usual blood tests but are improved with hydroxyurea.

In thirteen of the cases, we found data relating to cognitive status. The evaluation was conducted through a mini-mental state examination (MMSE) in four patients, with MoCA in one case. For the rest, there was no information regarding the type of evaluation that was employed [1,4,12,14,27]. Two of the patients were found to have mild cognitive impairment, one had moderate impairment, and all three had PV [15,28,29].

Two cases of PV-associated reversible behavioral changes with frontal lobe syndrome improved after PV-specific treatment [28,29]. The patients were not tested for the JAK2 mutation.

**Table 2:** Literature review of all of the MPD cases associated with chorea published in the last 40 years.

Reference	NOC	Age (y)	G	MPN	Comorbidities	Chorea Site	Other Signs or Symptoms	Chorea Onset	Organomegaly	Polycythemia	Thrombocythemia	Leukocytosis	HV	Bone Marrow Biopsy	Genetic Test for HD	JAK2 Mutation	Brain Imaging	Treatment	Clinical Follow-Up after Treatment	Mental Status
Coppack et al. [15]	1	57	F	Post PV MF and reverting to PRV (TMPD)	History of chorea relieved with chlordiazepoxide (St Vitus’ dance in 1920 following rheumatic fever), left ventricular hypertrophy	Generalized chorea and orofacial muscles	plethora; loose teeth, and mouth and tongue ulcers	N/A	Splenomegaly and hepatomegaly	Yes (Hb 21.8 g/dL)	No (400 × 10^9^/L)	Yes (18 × 10^9^/L)	Yes	Normal	N/A	N/A	CT: normal basal ganglia. Mild cerebral atrophy with small infarcts in the right cerebellum and left parietal lobe	Radiophosphorus, phlebotomies	Improvement of chorea, hematological abnormalities, and cognitive status	Memory deterioration and attacks of unresponsiveness of 20–30 s
Mas et al. [25]	2	72	F	PV	N/A	Generalized chorea and orofaciolingual muscles	Transient attacks of visual blurring and right hemiparesis, epistaxis	7Y	No splenomegaly	Yes (Hb 18.5 g/dL, RBCs 6 × 10^12^/L)	No (190 × 10^9^/L)	Yes (12.1 × 10^9^/L)	N/A	Global hyperplasia without fibrosis	N/A	N/A	CT: normal	Phlebotomies, haloperidol	No recurrence of polycythemia or neurological symptoms	Normal
		73	F	PV	N/A	Left limbs and orofaciolingual muscles	plethora; dysarthria, grimacing, grunting, transient attacks of dizziness, visual blurring, and epistaxis.	5M	No splenomegaly	Yes (Hb 18.5 g/dL, RBCs 6.6 × 10^12^/L)	No (372 × 10^9^/L)	No (10.7 × 10^9^/L)	N/A	Global hyperplasia without fibrosis	N/A	N/A	CT: normal	Phlebotomies, haloperidol	Remission of chorea	N/A
Cohen et al. [23]	1	65	F	PV	Hypertension and an ovarian tumor	Limbs and orofacial dyskinesia	plethora; dysarthria, dizziness, vertigo, and weight loss	N/A	Splenomegaly. No hepatomegaly	Yes (Hb 18.2 g/dL, Ht 58%)	No (450 × 10^9^/L)	No (11.6 × 10^9^/L)	Yes	Hypercellularity and normal chromosomal analysis	N/A	N/A	CT: normal	Phlebotomies	Resolution of chorea and blood abnormalities	N/A
Nazabal et al. [26]	1	74	F	PV	PV 10 y prior	Generalized chorea and orofaciolingual muscles	Moderately severe dysarthria, grimacing, and grunting	N/A	No splenomegaly	Yes (Hb 16.8 g/dL, RBCs 7.60 × 10^12^/L)	Yes (474 × 10^9^/L)	Yes (25 × 10^9^/L)	N/A	Global hyperplasia without fibrosis	N/A	N/A	MRI: normal	Phlebotomies, haloperidol	Improvement of chorea and normalization of Hb	Normal
Kumar et al. [11]	1	82	F	PV	Hypertension and smoking	Trunk, both upper limbs, tongue, lips, and posterior pharyngeal wall	plethora	A few days	No splenomegaly	Yes (Hb 18.1 g/dL, Ht 56%, RBCs 6.92 × 10^12^/L)	Yes (492 × 10^9^/L)	No (9.6 × 10^9^/L)	N/A	N/A	N/A	Positive	MRI: age-related cortical atrophy	Hydroxyurea (1000 mg per day)	Remission of chorea and improvement of hematological abnormalities	Normal
Huang et al. [30]	1	70	F	PV	N/A	Limbs and orofaciolingual muscle	Erythromelalgia of the hands with mild clubbing of the fingers	6M	No splenomegaly	Yes (Hb 16.8 g/dL, Ht 51.7%, RBCs 5.91 × 10^12^/L)	Yes (769 × 10^9^/L)	No	N/A	N/A	N/A	Positive	MRI: normal. SPECT reduced uptake in the bilateral basal ganglia, especially on the left side. FDG PET increased FDG uptake over the right dorsolateral prefrontal cortex and left insular cortex.	Phlebotomies	Improvement of chorea and hematological abnormalities	N/A
Ghorbel et al. [31]	1	78	M	PV	None	Limbs and face	Headache	1M	No splenomegaly	Yes (Hb 20 g/dL, Ht 62.3%)	No	No	N/A	Hypercellularity of the three lines	N/A	Positive	CT: normal	Hydroxyurea 200 mg OD	Resolution of chorea and improvement of hematological abnormalities	N/A
Severs et al. [28]	1	73	F	PV	Osteoporosis, recurrent urinary tract infections, and polymyalgia rheumatica	Upper limbs	plethora; positive snout, glabellar, right palmomental reflexes, and automatisms of the mouth and lips	N/A	No splenomegaly. No hepatomegaly	Yes (Hb 18.7 g/dL, Ht 57%)	No	No	N/A	N/A	N/A	Positive	MRI: mild ischemic white matter lesions	Phlebotomies	Improvement of chorea, hematological abnormalities, and psychological status	Mild cognitive impairment with frontal syndrome
Lew et al. [32]	1	87	F	PV	None	Left hemichorea and face	Dysarthria and facial asymmetry	N/A	No splenomegaly	No (Hb 15.6 g/dL, Ht 44.2%)	No (281 × 10^9^/L)	No (8.6 × 10^9^/L)	N/A	N/A (patient refused)	N/A	Positive	MRI/MRA: normal	Phlebotomy, hydroxyurea	Resolution of chorea improvement and hematological abnormalities	N/A
Bhargava et al. [24]	1	58	F	PV	None	Right hemichorea	None	N/A	Mild hepatomegaly and splenomegaly	Yes (Hb 17.5 g/dL)	No (305 × 10^9^/L)	Yes (13.48 × 10^9^/L)	N/A	Panmyelosis	BCR-ABL1- negative	Positive	MRI: symmetrical hyperintense in the basal ganglia on the T2-weighted and FLAIR sequence without any significant restriction on diffusion-weighted image or enhancement	Phlebotomies, hydroxyurea, low-dose aspirin	No recurrence of hemichorea	N/A
Venkatesan et al. [4]	1	55	F	ET	N/A	Limbs, face, and tongue	Dysarthria	N/A	No splenomegaly. No hepatomegaly	Yes (Hb 15.1 g/dL, Ht 46.3%, RBCs 5.07 × 10^12^/L)	Yes (1092 × 10^9^/L)	Yes (14.2 × 10^9^/L)	N/A	Hypercellularity with a marked increase in megakaryocytes	N/A	Positive	MRI: normal	Hydroxyurea	Resolution of chorea and improvement of hematological abnormalities	MMSE 28
Liu et al. [12]	1	70	F	PV	None	Limbs and orofaciolingual muscles	plethora; erythromelalgia, dysarthria, and dysphagia	4D	No splenomegaly	Yes (Hb 20.1 g/dL, Ht 65.8%)	N/A	N/A	N/A	Hypercellularity, erythroid hyperplasia, granulocyte/erythroid ratio 1:2, and megakaryocytic 8–10/higher power field	N/A	Positive	MRI: mild ischemic white matter lesions	Hydroxyurea 1.500 mg, Clopidogrel 50 mg per day	Resolution of chorea	MMSE 29
Degnan et al. [2]	1	84	M	PV	Dukes A sigmoid adenocarcinoma (treated with curative polypectomy) and benign prostatic hypertrophy	Limbs and axial, orofaciolingual muscles	Dysarthria and involuntary coughing and grunting	2M	No splenomegaly. No hepatomegaly	Yes (Hb 19.9 g/dL, Ht 58.6%)	Yes (352 × 10^9^/L)	Yes (21.2 × 10^9^/L)	N/A	N/A	Negative for Huntington’s	Positive	CT: small vessel disease	Phlebotomies	Resolution of chorea and hematological abnormalities	N/A
Rossi et al. [33]	1	72	F	PV	N/A	Right hemichorea and orofacial muscles	None	N/A	No splenomegaly. No hepatomegaly	Yes (Hb 18.5 g/dL, Ht 56%)	N/A	N/A	N/A	N/A	Negative for Huntington’s, C9orf72-negative.	Positive	MRI: normal	Phlebotomy, hydroxyurea	Resolution of chorea	N/A
Garcia-Cabo et al. [33]	1	96	M	PV	PV previously treated with hydroxyurea, hypertension, atrial fibrillation, and chronic lower limb ischemia	Left arm and face	Frontal lobe syndrome	4D	No splenomegaly	No (Hb 15.5 g/dL, Ht 46%)	No	No	N/A	N/A	N/A	N/A	CT: normal	Hydroxyurea	Improvement of chorea	Mild cognitive impairment with frontal syndrome
Rodrigues et al. [34]	1	75	F	PV	Myelodysplastic syndrome under investigation, depression, and hypertension	Limbs, mouth, and face	Erythromelalgia, generalized pruritus, weight loss of 16 kg over 6M, and mild rigidity of the limbs	3M	No splenomegaly. No hepatomegaly	Yes (Hb 17.4 g/dL)	Yes (600 × 10^9^/L)	No (9.5 × 10^9^/L)	N/A	Hypercellularity with erythroid and megakaryocytic predominance	Negative for Huntington’s	Positive	MRI: microangiopathic leukoencephalopathy	Risperidone, hydroxyurea	Improvement of chorea and hematological abnormalities	Normal
Bhattacharjee [1]	1	80	F	PV	Hypertension	Upper limbs and orofacial muscles	Motor impersistence evidenced by sustained tongue protrusion on request	6M	No splenomegaly. No hepatomegaly	Yes (Hb 17.6 g/dL, Ht 57%)	Yes (6.8 × 10^9^/L)	Yes (14 × 10^9^/L)	N/A	N/A	Negative for Huntington’s	Positive	MRI: normal	Phlebotomy, Aspirin 75 mg OD	No improvement of chorea and improvement of hematological abnormalities	MMSE 25
Koya et al. [14]	1	79	F	ET	ET treated with hydroxyurea 500 mg OD	Generalized chorea	Weight loss	1Y	No splenomegaly	No (Hb 9.1 g/dL, Ht 29.3%, RBCs 2.67 × 10^12^/L)	No (341 × 10^9^/L)	No (4.01 × 10^9^/L)	N/A	Mild dysplastic changes in the erythroid series	Negative for Huntington’s	Positive	MRI: small vessel disease and non-specific symmetric T2 and FLAIR hyperintensities within the brainstem	Tetrabenazine 12.5 mg twice daily	Improvement of chorea	MMSE 28
Bette et al. [18]	1	86	M	PV	Left amaurosis fugax, left acoustic neuroma treated with gamma knife radiation, and C2 to C7 spinal fusion complicated by left hemiparesis.	Generalized chorea	plethora; dysarthria, diffuse ecchymoses, left hemiparesis, involuntary facial grimacing, tongue chewing, grunting, shoulder shrugging, and 17-pound weight loss	1M	No splenomegaly. No hepatomegaly	No (Hb 14.4 g/dL)	No (209 × 10^9^/L)	Yes (16.4 × 10^9^/L)	N/A	N/A	Huntington- negative	Positive	MRI: left internal auditory canal enhancement	Hydroxyurea, tetrabenazine	Improvement of chorea and hematological abnormalities	N/A
De Lil et al. [27]	2	86	F	PV	None	Right-sided hemiballism	Psychotic derangement, aphasia, and apraxia	1Y	No splenomegaly. No hepatomegaly	Yes (Hb 16.1 g/dL, Ht 49%)	Yes (605 × 10^9^/L)	Yes (12.9 × 10^9^/L)	N/A	N/A	N/A	Positive	MRI: mild generalized atrophy and cerebral small vessel disease	Phlebotomies, hydroxycarbamide	Improvement of chorea, hematological abnormalities, and neuropsychiatric findings	MOCA 16
		71	M	PV	None	Right arm	Headaches, dizziness, double vision, and ataxia of the upper limbs	Several months	N/A	Yes (Hb 21.8 g/dL, Ht 67%)	Yes (404 × 10^9^/L)	No (7.3 × 10^9^/L)	N/A	N/A	Huntington—negative	Positive	MRI: insular venous developmental anomaly	Phlebotomies	Improvement of chorea and ataxia and resolution of headache and visual disturbances	N/A
Calculli et al. [13]	1	61	F	ET	ET treated with hydroxyurea, which was interrupted right before symptomatic onset	Left upper limb, and ipsilateral foot and toes, and oromandibular muscles	N/A	3M	N/A	N/A	Yes (816 × 10^9^/L)	N/A	N/A	N/A	Huntington—negative	Positive	MRI: chronic vascular infarction in the right caudate nucleus	Hydroxyurea 1000 mg OD, tetrabenazine 12.5 mg TD	Improvement of chorea and hematological abnormalities	N/A
Li et al. [35]	1	68	M	Post-PV MF	PV 10 y prior	Left hemichorea and orofaciolingual muscles	plethora; delirium, agitation, pouting, and grimacing	1D	No splenomegaly. No hepatomegaly	No (Hb 11.4 g/dL, Ht 41.9%, RBCs 4.98 × 10^12^/L)	No (125 × 10^9^/L)	Yes (36.5 × 10^9^/L)	N/A	Global hyperplasia with fibrosis, consistent with post-PV MF	N/A	Positive	MRI: encephalomalacia of the left frontal and right parietal lobes, gliosis, and cerebral small-vessel disease	Hydroxyurea, tiapride hydrochloride, clonazepam, quetiapine	Improvement of chorea and hematological abnormalities	Neuropsychological testing was not possible to complete

CT: computer tomography; D: days; G: gender; Hb: hemoglobin; HD: Huntington’s disease; Ht: hematocrit; HV: hyperviscosity; M: months; MRI: magnetic resonance imaging; N/A: not available; NOC: number of cases; OD: once daily; RBCs: red blood count cells; TMPD: transitional myeloproliferative disease.

## 4. Conclusions

To our knowledge, this is the first published case of JAK2-positive PMF associated with chorea and dementia, mimicking Huntington’s disease. The possibility of an associated unrelated major cognitive disorder cannot be ruled out at this stage. Still, the relatively simultaneous onset of dementia and chorea suggests a common mechanism. The profile of cognitive deficit did not suggest Alzheimer’s disease, meeting the criteria for frontotemporal dementia. Severe depression with suicidal attempts is again reminiscent of Huntington’s disease, which has some of the highest rates of suicide among the major cognitive disorders [36].

The chorea in our patient was not so severe as to require treatment, so he was discharged only with hydroxyurea and antidepressive treatment. At the 3-month, and 10-month visits, there were significant improvements in his chorea. This aspect is puzzling. Since our patient had normal cell blood counts, we cannot attribute any therapeutic response to the modification of blood viscosity and an interesting question arises whether the improvement is secondary to the hydroxyurea treatment and what the the mechanism is.

## Figures and Tables

**Table 1:** Timeline of the case report.

Test	August 2022 (Psychiatry Department)	September 2022 (Hematology Department)	November 2022 (Neurology Department)	February 2023 (Neurology Department)	September 2023 (Neurology Department)	Reference Values
Red cell count	5,630,000/mm^3^	5,680,000/mm^3^	5,240,000/mm^3^	5,410,000/mm^3^	4,800,000/mm^3^	4,500,000–6,200,000/mm^3^
Hemoglobin	15 g/dL	15.4 g/dL	15.2 g/dL	16.5 g/dL	14.5 g/dL	12.3–17 g/dL
Hematocrit	46.9%	47.9%	47.1%	50.8%	46.1%	37–52%
White cell count	4260/mm^3^	5140/mm^3^	5890/mm^3^	4800/mm^3^	3870/mm^3^	4000–10,000/mm^3^
Platelet count	252,000/mm^3^	305,000/mm^3^	252,000/mm^3^	200,000/mm^3^	174,000/mm^3^	150,000–450,000/mm^3^
INR	-	1.75	1.61	1.9	2.01	0.86–1.14
Clinical presentation	Severe depression Suicidal attempt Generalized chorea	Generalized chorea	Generalized chorea Hypersomnia Hyperphagia Cognitive decline	No choreiform movements at the time of examination (family members report noticing them occasionally)	No choreiform movements at the time of examination (family members report noticing them occasionally)	
Neurocognitive status	MMSE 15p	-	MOCA 19p	MOCA 16p	MOCA 17p	
Hydroxyurea treatment	Without treatment for approximately 7 years	Reinitialization of hydroxyurea 500 mg per day	Hydroxyurea 500 mg per day	Hydroxyurea 500 mg per day	Hydroxyurea 500 mg per day	

## Data Availability

No new data were created or analyzed in this study. Data sharing is not applicable to this article.
